# Interactions between Cancer-Associated Fibroblasts and T Cells in the Pancreatic Tumor Microenvironment and the Role of Chemokines

**DOI:** 10.3390/cancers13122995

**Published:** 2021-06-15

**Authors:** Laia Gorchs, Helen Kaipe

**Affiliations:** 1 Department of Laboratory Medicine, Karolinska Institutet, 14152 Stockholm, Sweden; 2 Clinical Immunology and Transfusion Medicine, Karolinska University Hospital, 14152 Stockholm, Sweden

**Keywords:** pancreatic ductal adenocarcinoma (PDAC), cancer-associated fibroblasts (CAFs), T cells, tumor microenvironment, immune checkpoint inhibitors, chemokines

## Abstract

**Simple Summary:**

Despite advances in therapeutic modalities, the five-year overall survival for pancreatic cancer is still less than 10%. Pancreatic tumors are characterized by a highly fibrotic stroma comprised of activated cancer-associated fibroblasts (CAFs) which surrounds the cancer cells. Pancreatic CAFs are involved in creating an immunosuppressive tumor microenvironment by secretion of immunoregulatory and chemoattractive factors, which prevent tumor-reactive T-cell responses. This review article discusses recent discoveries about the role of different subsets of CAFs as regulators of anti-tumor immunity in pancreatic cancer, with emphasis on chemokines and suppressive factors. Understanding the interactions between T cells and CAFs as well as their spatial distribution within the tumor is of great importance for the development of novel targeted therapies to overcome immunosuppression and to enhance immunotherapy.

**Abstract:**

Less than 10% of patients diagnosed with pancreatic ductal adenocarcinoma (PDAC) survive 5 years or more, making it one of the most fatal cancers. Accumulation of T cells in pancreatic tumors is associated with better prognosis, but immunotherapies to enhance the anti-tumor activity of infiltrating T cells are failing in this devastating disease. Pancreatic tumors are characterized by a desmoplastic stroma, which mainly consists of activated cancer-associated fibroblasts (CAFs). Pancreatic CAFs have emerged as important regulators of the tumor microenvironment by contributing to immune evasion through the release of chemokines, cytokines, and growth factors, which alters T-cell migration, differentiation and cytotoxic activity. However, recent discoveries have also revealed that subsets of CAFs with diverse functions can either restrain or promote tumor progression. Here, we discuss our current knowledge about the interactions between CAFs and T cells in PDAC and summarize different therapy strategies targeting the CAF–T cell axis with focus on CAF-derived soluble immunosuppressive factors and chemokines. Identifying the functions of different CAF subsets and understanding their roles in T-cell trafficking within the tumor may be fundamental for the development of an effective combinational treatment for PDAC.

## 1. Introduction

Pancreatic cancer is projected to be the second leading cause of cancer-related deaths in 2030 as a result of the lack of an effective treatment and the increasing incidence rate [1]. The only potential cure for pancreatic cancer is surgery, but due to its late detection only 15–20% of the diagnosed patients present with resectable tumors, and with surgery alone, less than 10% survive 5 years or more. Resection followed by chemotherapy increases the 5-year overall survival to only 16–20% [2,3]. The standard treatment for unresectable tumors is chemotherapy but the median overall survival is at best 16 months [4]. Therefore, there is a pressing need to find new therapies.

Although cancer immunotherapy has been shown to be effective against a variety of cancers during the last decade, there is very little progress in pancreatic cancer [5]. The majority of pancreatic tumors are defined as pancreatic ductal adenocarcinoma (PDAC), which is characterized by a dense stroma surrounding the cancer cells [6]. Activated cancer-associated fibroblasts (CAFs) represent the major cellular component in the pancreatic tumor stroma. Release of extracellular matrix components by CAFs triggers fibrosis which obstructs the intra-tumoral vessels and prevents therapy delivery and infiltration of tumor-reactive immune cells. Therefore, it is likely that immunotherapy combined with other treatments targeting the stromal barrier could be promising for pancreatic cancer patients.

CAFs release a number of different factors, including chemokines, cytokines, and growth factors, that promote immunosuppression through recruitment of immunosuppressive cells such as T regulatory cells (Tregs) and myeloid cells, upregulation of immune checkpoint molecules on T cells, and regulation of T-cell migration. It is still not well understood which factors are involved in regulating T-cell exhaustion and migration. However, several recent studies and subsequent clinical trials support that reprogramming of the suppressive microenvironment by blocking certain chemokine/chemokine receptor axes can improve immunotherapy outcomes in pancreatic cancer patients.

In this review, we will discuss the interactions between CAFs and T cells and explore therapeutic treatments that target the CAF–T-cell axis, with a focus on the role of immunosuppressive factors and chemokines.

## 2. Pancreatic Tumor Microenvironment

### 2.1. The Biology and Function of CAFs in Pancreatic Cancer

PDAC tumor nests are encapsulated by the desmoplastic stroma and CAFs can constitute up to 90% of the total tumor mass in PDAC. The main cellular source of CAFs are resident pancreatic stellate cells, but recruitment of mesenchymal stromal cells from the bone marrow has also been suggested to contribute to the fibroblastic stroma [7]. Under normal conditions, pancreatic stellate cells are in a state of quiescence and their main function is to maintain tissue homeostasis. In the presence of cancer cells or during injury, pancreatic stellate cells acquire an increased contractile ability similar to that in wound healing, which promotes the expression of α-smooth muscle actin (α-SMA) and desmin and loss of their characteristic cytoplastic lipid droplets [6,8]. Unlike wound healing, fibroblasts in tumors remain activated, which results in a pathological release of extracellular matrix components which triggers fibrosis [9]. It has been suggested that extracellular matrix stiffness plays a role in promoting cancer progression [10], and activated pancreatic stellate cells also stimulate angiogenesis and facilitate the invasion and extravasation of cancer cells [11].

### 2.2. Strategies to Target CAFs

Since the desmoplastic stroma has been suggested to play a tumor-supporting role and function as a physical barrier to delivery of chemotherapies to the tumor [12,13], attempts have been made to eradicate CAFs from tumors. However, recent studies and subsequent clinical trials suggest that local depletion or inhibition of CAFs is associated with increased tumor aggressiveness and progression rather than reduction [14,15,16]. Ablation of α-SMA^+^ cells in a murine PDAC model led to reduced desmoplasia, but the tumors were more aggressive and exhibited an undifferentiated phenotype, resulting in shorter survival [14]. Neoplastic cell deletion of Sonic Hedgehog 1, a major driver of the desmoplasic reaction, led to similar results in murine PDAC [16,17]. A clinical trial that combined chemotherapy with a Hedgehog inhibitor was terminated prematurely due to shortened patient survival [15]. Fibroblast activation protein (FAP) is expressed by the majority of pancreatic CAFs (90%) with a higher expression intensity on CAFs localized close to the tumor nests, and high FAP expression is associated with shorter overall survival [18]. Several in vivo studies have shown that FAP inhibition leads to reduced tumor progression by favoring immune control [19,20,21]. FAP inhibition enhanced the anti-tumor activity of immune checkpoint inhibitors [19], but it did not improve survival in a PDAC mouse model in one of the studies [20]. Adoptive transfer of chimeric antigen receptor (CAR) T cells directed to FAP inhibited pancreatic cancer cell growth [22], but infusion of FAP reactive CAR T cells has also been reported to trigger bone marrow toxicity and cachexia [23], a condition often seen in advanced PDAC.

Focal adhesion kinase (FAK1) is often overexpressed in PDAC and promotes tumor fibrosis, and is associated with low infiltration of effector T cells. Targeting FAK reduces fibroblast activation and decreases immunosuppressive cell infiltration [24]. The combination of FAK inhibitors and PD-1 blockade has shown promising synergistic effects in mouse models [24]. Several clinical trials targeting both FAK and PD-1 are currently ongoing (NCT02546531, NCT03727880, NCT02758587).

Another approach has been to reverse reactive CAFs towards a quiescent state. Vitamin D_3_ metabolites (1,25-dihydroxyvitamin-D_3_ or calcipotriol) have been shown to reverse activated CAFs to quiescent fibroblasts [25]. Activation of the vitamin D_3_ receptor on fibroblasts resulted in a reduction in pancreatic fibrosis and also increased the response to chemotherapy in a murine model. In vitro studies also suggest that calcipotriol promotes an anti-tumorigenic phenotype of CAFs, but that it also impairs T-cell-mediated immunity [26]. Ongoing clinical trials are investigating stroma remodeling combined with vitamin D_3_ and immunotherapy (NCT03331562, NCT03519308, NCT034415854).

Rho-associated protein kinase (ROCK) is an effector protein of the Rho GTPase family which is often overexpressed in pancreatic cancer [27]. ROCK regulates several cell functions, including cell contraction, cell adhesion, and cell migration, through the regulation of the cytoskeleton [27,28]. ROCK inhibitors have been shown to reduce CAF activation by reducing α-SMA and collagen I expression, leading to an enhanced gemcitabine delivery and improved survival [27]. In line with this, ROCK inhibitors have been also shown to reduce extracellular matrix deposition by CAFs, which led to an impaired cancer cell invasion and increased response to gemcitabine in in vitro 3D models and mouse models [29,30].

A prodrug of the plant-derived chemotherapeutic substance triptolide, Minnelide, has been shown to inactive CAFs and promote tumor regression in a TGF-β-dependent manner in a preclinical model [31]. Furthermore, Minnelide reduced extracellular matrix contents in the stroma which led to improved vascular patency and a more efficient delivery of standard of care chemotherapy [32]. To summarize, these studies highlight the importance of remodeling activated CAFs to a quiescent state to improve the delivery and efficacy of standard therapies.

### 2.3. CAF Heterogeneity

**Figure 1:**
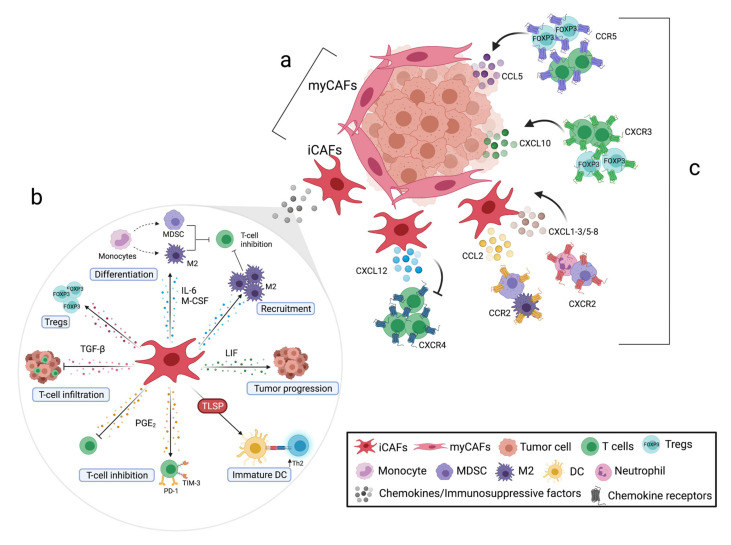
Schematic representation of the immunoregulatory functions of cancer-associated fibroblasts (CAFs) and chemokines on T cells in the pancreatic tumor microenvironment. (**a**) Two different subsets of CAFs with diverse functions have been identified in pancreatic cancer: myofibroblastic (myCAFs), which are located close to the tumor nests and likely suppress tumor cell growth, and inflammatory CAFs (iCAFs) which are located more distantly from the tumor nests and secrete inflammatory factors with pro-tumorigenic functions. (**b**) The pro-tumorigenic factors can regulate the differentiation, migration, and function of myeloid cells such as myeloid-derived suppressor cells (MDSC), M2 macrophages, and dendritic cells (DC) which in turn inhibit T-cell migration, activation, proliferation, and differentiation. CAFs also disrupt T-cell functionality by promoting expression of immune checkpoint inhibitors (PD-1, TIM-3), restricting T-cell infiltration into the tumor nests, and promoting T regulatory cells (Tregs). (**c**) CAFs can entrap T cells in the tumor stroma through the CXCL12–CXCR4 axis and recruit MDSCs, M2-type macrophages, and neutrophils through the CCL2–CCR2 axis and CXCR2 ligation. Other chemokines in the tumor microenvironment such as CXCL10 and CCL5 can have a dual role by promoting the infiltration of T cells but also of Tregs. Abbreviations: M-CSF, macrophage colony-stimulating factor; LIF, leukemia inhibitory factor; TSLP, thymic stromal lymphopoietin; PGE_2_, prostaglandin E_2_; TGF-β, transforming growth factor β. Figure created with BioRender.com.

One plausible explanation for the findings that CAFs acts to restrain rather than support tumor cell growth and invasion is that there are different subpopulations of CAFs in the tumor stroma with diverse functions. Öhlund et al. identified two phenotypically and functionally distinct CAF subsets within pancreatic tumors [33]. Myofibroblastic CAFs (myCAFs) expressing high levels of α-SMA are generally localized in close proximity to the tumor nests, whereas inflammatory CAFs (iCAFs) are positioned more distantly from the malignant cells in the desmoplastic stroma (Figure 1a). iCAFs secrete an array of inflammatory mediators with pro-tumorigenic functions, such as interleukin (IL)-6, IL-8, leukemia inhibitory factor (LIF), CCL2, and CXCL2. IL-1α was shown to induce iCAFs by downstream JAK/STAT activation, whereas tumor-derived TGF-β and SMAD2/3 signaling counteracts this process, resulting in differentiation into myCAFs [34]. Both subsets can dynamically revert from one phenotype to another, suggesting that CAFs display plasticity based on spatial location and microenvironmental factors [33]. Another study based on microarray data revealed that the pancreatic tumor stroma can be divided into normal and activated subtypes, where normal stroma was associated with better prognosis [35]. Normal stroma was characterized by high expression of the genes encoding α-SMA, vimentin, and desmin, whereas the activated subtype was characterized by high expression of FAP and genes associated with macrophages.

Single-cell RNA sequencing has further revealed several subsets of CAFs in PDAC [36,37,38,39] and other types of cancer [40,41]. A population of CAFs with antigen-presenting capacities (apCAF) expressing MHC class I-related genes could activate CD4^+^ T cells in an antigen-specific manner, but they lacked the expression of co-stimulatory markers suggesting that they would fail to prime a naïve T-cell response [37]. However, another study suggested that this subset of cells were mesothelial cells that had acquired expression of fibroblast genes in the tumor microenvironment [36]. It was recently shown that inhibition of Hedgehog signaling alters CAF composition, with a reduction in myCAF numbers and an increase in iCAF numbers [42]. This was also correlated with a decrease in cytotoxic T cells and expansion in regulatory T cells and PD-L1^+^ macrophages, suggesting that enrichment of iCAFs is associated with immunosuppression. A recent study by Chen et al. showed that deletion of type I collagen in α-SMA^+^ myCAFs aggravates pancreatic tumor progression in a murine model [38], supporting that myofibroblast-derived fibrillar proteins act to prevent tumor advancement. Thus, current knowledge suggests that myCAFs restrain tumor cell growth, whereas iCAFs display a more pro-tumorigenic function by secretion of inflammatory factors that promote tumor growth.

CAF heterogeneity can also be induced by the genetic status of the tumor suppressor gene p53 in pancreatic tumor cells. A study using a genetically engineered pancreatic cancer mouse model showed that cancer cells with a gain-of-function mutant p53 gene (GOF p53) promoted an aggressive CAF phenotype, resulting in an increased expression of contractile markers as compared to CAFs cultured with p53 null mutant cancer cells (p53 null) [43]. Moreover, CAFs educated by GOF p53 cancer cells promoted invasion of the p53 null cancer cells to the same extent as the highly invasive GOF p53 cancer cells. In the same way, CAFs cultured with p53 null cancer cells can adopt an aggressive phenotype when cultured with GOF p53 tumor cells, further supporting that CAFs display plasticity. The authors identified an extracellular matrix proteoglycan, perlecan, secreted by GOF p53 educated CAFs, as a key factor for promoting a permissive environment for cancer cell invasion and metastasis in vivo [43]. Exosomes derived from mutant-p53-expressing tumor cells also confer stromal architecture remodeling by affecting normal fibroblasts in the microenvironment to deposit a pro-invasive extracellular matrix, which can pave the way for metastasis [44].

### 2.4. Tumor-Infiltrating Lymphocytes

T cells have the capacity to recognize and kill tumor cells, but malignant cells can evade immune surveillance by inducing T-cell exhaustion. Pancreatic tumors have generally been considered as immune-privileged in nature, but accumulation of CD8^+^ cytotoxic T cells in the tumor is correlated with a better prognosis in PDAC [45,46]. The use of immune checkpoint inhibitors has provided a paradigm shift in the treatment of some malignancies, including melanoma [47]. These include antibodies directed to the co-inhibitory markers PD-1, PD-L1, and CTLA-4, which can allow T cells to regain their function and mediate killing of tumor cells [48]. In PDAC, blockade of co-inhibitory receptors has so far been unsuccessful. The mutational burden in PDAC is low, which leads to poor antigenicity, as reflected by little expression and presentation of neoantigens which potentially can be detected by T cells as foreign. Melanoma and other types of cancer that are responsive to immune checkpoint inhibition are associated with high mutational burden [49], and there is a positive correlation between objective response rate to PD-1 inhibition and tumor mutational burden in multiple cancer types [50]. Nevertheless, it has been demonstrated that clonally expanded T cells with tumor specificity are present in pancreatic tumors [51], suggesting that T-cell responses to PDAC tumors should be feasible but that it may be limited by the microenvironment in the pancreas.

A novel treatment with the poly(ADP-ribose) polymerase (PARP) inhibitor, olaparib, has recently been approved by the FDA as a first-line maintenance treatment in BRCA-mutated metastatic pancreatic cancer. PARP inhibitors block the DNA repair machinery and may thus enhance cell death and chemotherapy efficacy. A randomized phase III clinical trial showed that patients who received olaparib had a median progression-free survival of 7.4 months compared to 3.8 months in the placebo group [52]. Due to their role in preventing DNA repair, PARP inhibitors may contribute to an increased tumor mutational burden and thus contribute to augmented anti-tumor T-cell responses [53]. Ongoing clinical trials are exploring the synergistic effects of immune checkpoint inhibitors and PARP inhibitors in several cancers, including pancreatic cancer (NCT02660034) [53].

Several studies suggest that the majority of T cells are entrapped in peritumoral stromal areas of pancreatic cancer with little infiltration in tumor nests [45,54,55,56,57]. The reason for this is not entirely clear, but it could be due to CAF-mediated retainment of immune cells [51,58] and due to an influx of suppressive Tregs and myeloid cells, such as myeloid-derived suppressor cells (MDSCs) and tumor-associated macrophages. PDAC-infiltrating T cells readily express co-inhibitory markers, including PD-1, LAG-3, and TIGIT [59], and it has been suggested that CAFs contribute to their exhausted phenotype [55]. Wartenberg et al. performed an integrated genomic and immunophenotypic classification of PDAC which displayed different subtypes with prognostic value [60]. The “immune escape” subtype, which was associated with poor outcome and occurred in more than half of the patients, contained few T cells and B cells but was enriched in FOXP3^+^ Tregs. This subtype also displayed a high-grade tumor budding, which is characterized by de-differentiated tumor cells dispersed as single cells or small clusters of tumor cells within the stroma. Tumor budding is associated with epithelial-to-mesenchymal transition, metastasis, and reduced patient survival [61]. It was recently shown that the number of both tumor- and stroma-infiltrating CD4^+^ and CD8^+^ T cells were reduced in pancreatic tumors with high tumor budding [62].

Other spatial computational studies in PDAC showed higher numbers of CD8^+^ T cells together with Tregs, MDSC, neutrophils, and TAMs at the tumor margins compared to the tumor center [63]. Importantly, higher CD8^+^ T-cell density in the tumor center was associated with prolonged patient survival. An immune profile including high M2 macrophages and neutrophils with low M1 macrophages was correlated with shorter overall survival, whereas high CD4^+^ and CD8^+^ T cells together with low Tregs was associated with longer overall survival in PDAC [64]. Similarly, a high CD8^+^/Tregs ratio was correlated with longer overall survival in another computational analysis in PDAC patients [65]. A recent study found that high CD4^+^/CD3^+^ ratio together with a low α-SMA/vimentin ratio on CAFs was correlated with shorter overall survival in pancreatic cancer of the body and tail [66].

Combining both quantification and localization of T cells within the tumor could provide promising prognostic tools for predicting survival in pancreatic cancer. However, further studies assessing the prognostic value of the spatial distribution of T cells in combination with different CAF subtypes in pancreatic cancer would be of great interest for better personalized combination therapies.

## 3. CAFs Modulate T-Cell Function in the Pancreatic Tumor Microenvironment

In order to reach and eradicate the tumor cells, effector T cells infiltrating the pancreas not only need to overcome the dense fibrotic barrier, but also the suppressive CAF secretome (Figure 1b). Activated CAFs contribute to immune evasion through the release of suppressive factors, chemokines, and expression of immune checkpoint ligands that can directly or indirectly, through the modulation of antigen-presenting cells, hamper T-cell effector functions. Table 1 summarizes immunological effects after targeting stromal-derived factors in preclinical models.

**Table 1:** Immunosuppressive targets in the pancreatic tumor microenvironment used in preclinical models and clinical trials with the reported observations on the effects on immune cells and the primary end point of the clinical trial.

Target	Observations in Preclinical Models [ref]	CLINICAL TRIALS					
		NCT	Treatment	Phase	Condition	Status	Primary Endpoint// Observations [ref]
IL-6							
		NCT00841191	Siltuximab	I/II	Unresectable	Completed	CBR// No benefit =inflammatory cytokines =Angiogenesis markers ↓pSTAT3 [67]
		NCT02767557	Tocilizumab Gemcitabine Nab-paclitaxel	II	Unresectable	Recruiting	OS
IL-6 + ICI							
	↓Tumor growth ↑Survival ↑T-cell infiltration [68]	NCT04258150	Nivolumab Ipilimumab Tocilizumab SBRT	II	Unresectable	Active	ORR
		NCT04191421	Siltuximab Spartalizumab	I/II	Unresectable	Recruiting	Determine dose
COX-2							
		NCT00176813	Celecoxib Gemcitabine Cisplatin	II	Unresectable	Completed	OS// No benefit [69]
			Celecoxib Gemcitabine	II	Unresectable	Completed	DFS/OS/tolerability// No benefit ↓VEGF [70]
			Celecoxib Gemcitabine	II	Unresectable	Completed	Toxicity/ORR// ↑OS ↓CA19.9 [71]
			Celecoxib Gemcitabine Irinotecan	II	Unresectable	Completed	Toxicity/ORR// ↑OS ↓CA19.9 [72]
		NCT03838029	Etodolac Propranolol Placebo	II	Resectable	Recruiting	DFS/biomarkers in blood
		NCT03498326	Celecoxib Gemcitabine	II	Resectable	Recruiting	DFS
COX-2 + ICI							
	↓Tumor growth ↑CD8^+^ T-cell infiltration [73]	NCT03878524	Multiple drugs including Celecoxib Nivolumab	II	Unresectable	Recruiting	Find the best combination of drugs
TGF-β							
		NCT00844064	AP 12009	I	Unresectable	Completed	MTD// ↑OS
		NCT04624217	SHR-1701	I/II	Unresectable	Recruiting	RP2D/ORR
		NCT03666832	TEW-7197	I/II	Unresectable	Recruiting	DFS
		NCT03685591	PF-06952229 Enzalutamide	I	Unresectable	Recruiting	DLT
TGF-β + ICI							
	↓Tumor growth ↑T-cell infiltration ↑CD8^+^ T-cell cytotoxicity [74,75]	NCT02734160	Galunisertib Durvalumab	I	Unresectable	Completed	DLT// Limited effects [76]
		NCT04429542	BCA101 Pembrolizumab	I	Unresectable	Recruiting	Safety/tolerability/DLT
		NCT02947165	NIS793 PDR001	I	Unresectable	Active	DLT

ref, reference; ICI, immune checkpoint inhibitor; NCT, clincialtrials.gov identifier; CBR, clinical benefit response; OS, overall survival; ORR, objective response rate; DSF, disease-free survival; MTD, maximum tolerated dose; RPD2, recommended phase 2 dose; DLT, dose-limiting toxicities; CA19.9, carbohydrate antigen; =, no changes; ↓, decrease; ↑, increase; //, separation between primary endpoint and observations.

### 3.1. Modulatory Functions of Pancreatic CAFs in Myeloid Cells

Priming of tumor-specific CD8^+^ T cells and CD4^+^ T helper (Th) 1 cells is key to mounting an effective immune response. However, CAFs can jeopardize the presentation of tumor-associated antigens to T cells by modulating the function and maturation of MDSCs, macrophages, and dendritic cells (DCs), and thus, suppress adaptive Th1 and cytotoxic immune responses.

Paracrine signaling by CAFs can promote the differentiation of monocytes and granulocytes to MDSCs. A study by Mace et al. showed that IL-6 released by primary pancreatic CAFs induced differentiation of peripheral blood mononuclear cells into functional MDSCs via STAT3 activation, which in turn suppressed T-cell proliferation [77]. Three-dimensional cultures of monocytes with spheroids containing pancreatic tumor cells and fibroblasts have been shown to increase the expression of immunosuppressive cytokines, such as IL-6 and macrophage colony-stimulating factor (M-CSF), known to induce MDSCs and M2-like macrophages [78]. M2 macrophages are low-efficiency antigen-presenting cells and their immunosuppressive activity in the tumor microenvironment is well established. CAF-derived IL-6 and M-CSF have been shown to directly promote the polarization of M2 macrophages in two different in vitro studies [78,79]. Consequently, differentiated M2 macrophages inhibit T-cell migration, activation, and proliferation within the tumor microenvironment and thus support PDAC progression [77,80,81]. Taken together, these findings show that CAF-derived IL-6 plays a key role in modulating the immune cell population towards a suppressive phenotype. Importantly, IL-6 has been shown to be mostly expressed by the stroma and not by the tumor cells [68]. Combined blockade of IL-6 and PD-L1 in mice models of pancreatic cancer led to attenuated tumor growth, prolonged survival, and increased infiltration of T cells [68].

Along with IL-6, CAF-derived LIF has also been identified as a major promoter of suppression in the tumor microenvironment. This member of the IL-6 family is also aberrantly expressed in pancreatic CAFs, in iCAFs in particular [26,33,82], and is correlated with poor prognosis [83]. We have shown that vitamin D_3_ metabolites altered the immunosuppressive CAF secretome and downregulated LIF secretion [26]. Moreover, experimental models of pancreatic cancer have shown that CAF-derived LIF activated pancreatic cancer cells and that it was correlated with tumor progression [82]. Interestingly, in glioblastoma models, LIF promoted the recruitment of M2 macrophages and the silencing of CXCL9, a cytokine that recruits T cells into the tumors [84]. These studies identify LIF as a promising therapeutic target in pancreatic cancer, but further studies evaluating CAF-mediated T-cell inhibition in response to LIF are necessary.

DCs are the most efficient antigen-presenting cells for priming naïve tumor-specific T cells to induce proliferation and activation and, thus, to drive Th1 differentiation. However, as shown in PDAC mice models, DCs in the tumor milieu and in the tumor-draining lymph nodes can be very few and express low costimulatory and maturation markers, impairing T-cell priming [85]. Studies showing a direct impact of CAFs on DCs in pancreatic cancer are scarce. However, an in vitro study suggested that DCs cultured in CAF supernatants adopt a suppressive phenotype in a thymic stromal lymphopoietin (TSLP)-dependent manner and promote Th2 differentiation of naïve CD4^+^ T cells [86]. Another study with hepatocellular CAFs showed that IL-6 induced the differentiation of regulatory DCs through the upregulation of IDO, which resulted in low costimulatory molecules expression, disabling T cells’ functions and inducing Tregs’ expansion [87].

Taken together, these studies suggest that targeting the stromal signaling may reduce myeloid-mediated suppression of anti-tumor T-cell activity and improve the efficacy of immune checkpoint therapeutics. Furthermore, the factors identified as the main drivers of immunosuppression in this context are produced in high levels from iCAFs, suggesting that this subset of CAFs play an important role in modulating myeloid and antigen-presenting cells in the tumor microenvironment.

### 3.2. Modulatory Functions of Pancreatic CAFs in T Cells

T-cell dysfunction in the tumor microenvironment comes with the expression of immune checkpoint molecules which triggers loss of proliferative and cytotoxic capacity upon binding to their ligands. The upregulation of immune checkpoints on T cells occurs due to a chronic tumor antigen stimulation and the exposure to suppressive cytokines. Significantly, co-expression of multiple co-inhibitory markers is associated with a worse functionality [88,89].

Pancreatic CAFs express some immune checkpoints, including PD-L1 and PD-L2 [55], and PD-L1/L2 overexpression in PDAC has been correlated with poor prognosis [90,91,92]. Even though it is well established that cancer cells upregulate these ligands as a mechanism of tumor immune evasion [93], very few studies have explored the immunomodulatory consequences of these ligands on CAFs. We and others have previously shown that blockade of PD-L1/L2 can partially restore CAF-mediated T-cell suppression [55,94]. However, further studies are needed to evaluate the direct interactions between CAFs and T cells in this context.

Besides immune checkpoints, CAFs can also disrupt T-cell functionality through the secretion of soluble factors. Prostaglandin E_2_ (PGE_2_) is the main metabolite generated by the enzyme cyclooxygenase 2 (COX-2), which is often overexpressed in the stroma of pancreatic cancer [95,96]. We have previously shown that PGE_2_ secreted by primary pancreatic CAFs inhibit T-cell proliferation and contribute to an upregulation of the immune checkpoint markers TIM-3 and PD-1 on activated T cells [55]. Another study found that COX-2 knockdown in tumor cells suppressed tumor growth and increased the number of tumor-infiltrating cytotoxic CD8^+^ T cells, which led to an improvement in immunotherapy in pancreatic mouse models [73]. This has also been demonstrated in experimental models of ovarian and colon carcinomas, which showed that the COX-2/PGE_2_ axis excludes T cells from the tumor milieu and that blockade of COX-2 or PGE_2_ increases the number and the cytotoxic effects of CD8^+^ T cells, which boosted the efficacy of anti-PD-1 therapy [97,98].

TGF-β is a pleiotropic cytokine and a major contributor to immunosuppression in the tumor microenvironment. Pancreatic CAFs express high amounts of TGF-β [8,96], which has been associated with tumor cell growth and extracellular matrix deposition [31,99]. The role of TGF-β in disabling the cytotoxic activity of T cells has been extensively studied [100,101]. Moreover, recent studies with other tumor types have also reported that CAF-derived TGF-β contributes to immune evasion by restricting T-cell infiltration [102,103,104,105]. However, studies in pancreatic cancer showing how CAF-derived TGF-β affects T cells are few. A recent study by Dominguez et al. identified a TGF-β–CAF subset associated with poor response to immune checkpoint blockade therapies [36]. Interestingly, two recent studies using experimental models showed that inhibition of TGF-β reduced CAF activation, resulting in reduced fibrosis and increased T-cell infiltration, which in turn improved PD-1 and PD-L1 treatment by enhancing CD8^+^ T-cell cytotoxicity [74,75]. Taken together, these findings suggest that blockade of TGF-β in pancreatic cancer could be promising for enhancing immune checkpoint therapies.

CAFs can also regulate the activity and the phenotype of other T-cell subsets in the tumor microenvironment, such as Tregs, natural killer (NK) cells, and γδ T cells. In vitro functional assays have shown that CAFs increase the proportion of FOXP3^+^CD4^+^ T cells [55]. Similarly, CAFs derived from other tumor types also promote the expression and recruitment of FOXP3^+^CD4^+^ T cells into the tumor milieu [41,106,107]. Importantly, increased numbers of Tregs are associated with reduced survival in pancreatic cancer [108]. Another study showed that CAFs can inhibit NK-cell cytotoxic activity [109]. However, the study did not assess which factors were responsible for the modulation of the NK phenotype.

In PDAC, γδ T cells might have a pro-tumoral activity by inhibiting T-cell responses [110]. A recent study by Seifert et al. showed a high correlation between the presence of γδ T cells and fibrosis. Moreover, it was found that γδ T cells were in close proximity to pancreatic CAFs and that they promoted the expression of IL-6 in CAFs [111]. To our knowledge, there are no studies showing the effects of CAFs on γδ T cells, but it is possible that there is a bidirectional interaction.

In conclusion, CAFs modulate T-cell effector functions by multiple mechanisms. Disrupting CAF-mediated signaling in the tumor microenvironment is a promising therapeutic strategy to boost the efficacy of immunotherapies in pancreatic cancer. However, it may be of great importance to target particular subsets of CAFs, such as iCAFs and TGF-β–CAFs, in order to achieve the desired effect.

## 4. Therapeutic Treatments to Target CAF-Derived Immunosuppressive Factors

Several clinical trials have evaluated the benefit of targeting immunosuppressive factors in pancreatic cancer patients measured by clinical outcomes. However, to our knowledge, there are no studies investigating the effects on the immune profile after therapy. Table 1 includes a summary of the completed and active clinical trials targeting CAF-derived immunosuppressive factors in pancreatic cancer.

A phase I/II clinical trial (NCT00841191) assessing the safety and efficacy of anti-IL-6, siltuximab, administered as a monotherapy to patients with pancreatic cancer, showed a good tolerance, but did not detect any clinical benefit [67]. The efficacy of anti-IL-6 combined with immune checkpoint inhibitors or with chemotherapy is currently being studied in several clinical trials (NCT04258150, NCT04191421).

The benefits of the COX-2 inhibitor, celecoxib, administered in combination with standard chemotherapy treatment, have been studied in several phase II clinical trials [69,70,71,72]. The treatment was well tolerated by the patients in all the studies but with varying clinical effects. In two of the trials, the COX-2 inhibitor did not demonstrate any significant clinical improvement [69,70]. However, two other clinical trials showed that the administration of COX-2 inhibitors partially improved the clinical outcomes. One study reported an overall clinical benefit rate of over 50% but the median survival was 9 months [71]. Another study showed a 4-fold increase in one-year overall survival for patients treated with combination therapy compared to chemotherapy alone [72]. The benefits of COX-2 inhibitors are being further investigated in several clinical trials (NCT03838029, NCT03498326, NCT03878524).

A phase I clinical trial (NCT02734160) evaluating anti-TGF-β-R1 combined with anti-PD-L1 in metastatic pancreatic cancer patients showed limited clinical effects with an objective response rate of only 3% and a median overall survival of 5 months [76]. The synergistic effect of anti-TGF-β and immune checkpoint inhibitors is being evaluated in different ongoing clinical trials (NCT04624217, NCT04429542, NCT02947165). Furthermore, a phase I/II clinical trial (NCT00844064) with advanced pancreatic cancer patients who received the TGF-β2 anti-sense oligonucleotide, OT-101, followed by subsequent chemotherapy, showed an improved overall survival [112]. Further clinical trials with anti-TGF-β are ongoing (NCT03666832, NCT03685591).

## 5. The Role of Chemotactic Factors in Pancreatic Tumor Immune Cell Infiltration

The spatial distribution of tumor-reactive immune cells in the tumor microenvironment is of great importance for efficient tumor eradiation. Since most T cells are localized in the desmoplastic stroma of the PDAC tumor [45,54,55,56], means to increase their mobility to reach the malignant cells could be crucial. Chemokines are low-molecular-weight proteins with chemoattractive capacities that, after signaling through their cognate receptors, promote cell migration towards and within tissues. The putative role of chemokines in the localization of immune cells within the tumor is only starting to be unraveled, but several recent studies point to the fact that chemokines can stimulate recruitment of both immunosuppressive and tumor-reactive immune cells into the tumor microenvironment (Figure 1c). Furthermore, accumulating data suggest that chemokines may play a key role in regulating immune cell infiltration and access to the tumor nests. Table 2 summarizes the outcomes of targeting stromal-derived chemokines in preclinical models.

**Table 2:** Inhibitors of chemokines used in preclinical models and clinical trials with the reported observations on the effects on immune cells and the primary endpoint of the clinical trials.

Target	Observations in Preclinical Models [ref]	CLINICAL TRIALS					
		NCT	Treatment	Phase	Condition	Status	Primary Endpoint// Observations [ref]
CCR2							
	+ CXCR2 target: ↓ MDSC infiltration [113]	NCT01413022	PF-04136309 Folfirinox	Ib	Unresectable	Completed	Optimal dose and toxicity// ↓TAMs ↑CD8^+^ and CD4^+^ T-cell infiltration [114]
		NCT02732938	PF-04136309 Gemcitabine Nab-paclitaxel	Ib/II	Unresectable	Completed	DLT// No benefit Pulmonary toxicity [115]
CCR5 + ICI							
		NCT04721301	Maraviroc Nivolumab Ipilimumab	I	Unresectable	Active	Safety and tolerability
CCR2 + CCR5 + ICI							
		NCT03184870	Multiple drugs including BMS813160 Nivolumab	I/II	Unresectable	Active	Toxicity/Tregs numbers/ORR/PFS
CXCR1/2 + ICI							
	↑ CD4^+^ and CD8^+^ T-cell infiltration [116,117] ↑ CD4^+^ and CD8^+^ T-cell cytotoxicity [116] ↓Neutrophils [116] ↓Metastasis ↓Tregs [117]	NCT04477343	SX-682 Nivolumab	I	Unresectable	Recruiting	MTD
CXCL12/CXCR4 axis							
		NCT02179970	AMD3100	I	Unresectable	Completed	Safety// ↑ T-cell, NK-cell infiltration and activation ↑ B-cell activation ↓CXCL8 [118]
CXCL12/CXCR4 axis + ICI							
	↑CD8^+^ T-cell infiltration and cytotoxicity [51]	NCT03168139	NOX-A12 Pembrolizumab	I/II	Unresectable	Completed	Safety// Stable disease ↑Th1 cytokines [119]
		NCT02826486	BL-0840 Pembrolizumab	IIa	Unresectable	Completed	ORR// ↑OS ↑CD8^+^ T-cell infiltration ↓MDSC ↓Tregs [120]
		NCT04177810	AMD3100 Cemiplimab	II	Unresectable	Recruiting	ORR
		NCT02907099	BL-0840 Pembrolizumab	II	Unresectable	Active	ORR
		NCT04543071	BL-0840 Cemiplimab Gemcitabine Nab-paclitaxel	II	Unresectable	Recruiting	ORR

ref, reference; NCT, clinicaltrials.gov identifier; ↓, decrease; ↑, increase; MDSC, myeloid-derived suppressor cells; TAM, tumor-associated macrophages; ICI, immune checkpoint inhibitor; NK, natural killer cells; Th1, T helper type 1 cells; OS, overall survival; DLT, dose-limiting toxicities; ORR, objective response rate; PFS, progression-free survival; MTD, maximum tolerated dose; // separation between primary endpoint and observations.

### 5.1. The Role of the CXC12/CXCR4 Axis in T-Cell Retention and Tumor Growth

Chemotactic factors can stimulate recruitment of immune cells to the tumor microenvironment via engagement with their corresponding receptors. However, certain chemokines have also been suggested to retain active immune cells in the stromal compartment. In a murine PDAC model, it was shown that CAFs prevent CD8^+^ T cells from reaching the tumor cells, a mechanism mediated by production of CXCL12 that retains CD8^+^ T cells in the stroma via CXCR4 ligation [58]. In line with this, Biasci et al. showed that CXCL12 suppresses directed migration of human immune cells towards other chemokines, including CXCL10 and CXCL16 [118]. iCAFs express higher levels of CXCL12 compared to myCAFs [33], suggesting that iCAFs may play a more prominent role in preventing T cells from entering the tumor nests. Feig et al. identified FAP^+^ CAFs as the main source of CXLC12 [19]. Blockade of CXCR4 led to an increased accumulation of T cells in a PDAC tumor model which synergized with blockade of PD-L1 [19]. The combined blockage of CXCR4 and PD-1 has also been shown to lead to an increased migration of T cells from the stroma into cancer-cell-rich regions in in vitro organotypic models of pancreatic cancer [51]. Perivascular CAF-derived CXCL12 is also implicated in attracting CXCR4^+^ macrophages toward blood vessels, which in turn leads to tumor cell intravasation in murine models [121]. It was further shown that radiation exposure increases secretion of CXCL12 from CAFs, which in turn promotes pancreatic cancer cell epithelial-to-mesenchymal transition and invasion in vitro and metastasis in vivo in a CXCL12–CXCR4-dependent manner [122]. PDAC-derived exosomes secreting macrophage migration inhibitory factor (MIF), another CXCR4 ligand with chemokine-like functions, has been suggested to be involved in initiating pre-metastatic niche formation in the liver in PDAC [123]. Thus, CXCR4 and its ligands not only prevent T cells from migrating from the stroma to the malignant cells, but also appear to be involved in tumor cell migration and invasiveness.

### 5.2. CXCR2 and CCR2 and Their Ligands Promote Infiltration of Suppressive Myeloid Cells

CXCR2 is the receptor for the chemokines CXCL1, CXCL2, CXCL3, CXCL5, CXCL6, CXCL7, and CXCL8 in humans. The primary immune function of CXCR2 is to regulate neutrophil migration from the bone marrow and recruitment to inflammatory sites, but recent studies also suggest that CXCR2 is involved in tumor progression by promoting accumulation of MDSCs, neutrophils, and other suppressive cells in pancreatic tumors [38,116,117]. Inhibition of CXCR2 abrogates tumorigenesis and metastasis in murine models and was also associated with an increased infiltration of T cells [116,117]. Furthermore, combined blockage of CXCR2 and PD-1 resulted in an improved animal survival [117]. In line with this, deletion of type I collagen in α-SMA^+^ CAFs was associated with an increased production of CXCL5 and subsequent influx of CD206^+^ARG1^+^ MDSCs into the tumor microenvironment [38]. Inhibition of CXCR2 and CCR2 in this model reversed infiltration of MDCSs and tumor progression and increased T-cell influx. CAFs have also been shown to express CXCR2 in PDAC and it was recently shown that macrophage-derived CXCL3 promotes differentiation of α-SMA^+^ CAFs by CXCR2 ligation [124]. CXCL3-primed CAFs promoted cancer metastasis and expression of CXCL3 was correlated with poor patient survival. To summarize, CXCR2 and its ligands promote recruitment of immunosuppressive myeloid cells with a concomitant decrease in T-cell infiltration. However, CAR T cells modified to express CXCR2 showed persistence in tumors and complete tumor regression in murine models of pancreatic cancer [125], suggesting that tumor-reactive immune cells can be modified to take advantage of the CXCR2 ligands in the tumor.

As with CXCR2, the CCR2/CCL2 axis is also involved in recruiting myeloid cells to the tumor microenvironment. It plays a particularly important role in attracting monocytes, which, after interactions with tumor- and stromal-derived factors, differentiate into suppressive tumor-associated macrophages at the site [126]. A combined blockage of CCR2 and CXCR2 in a murine PDAC model prevented both CCR2^+^ macrophages and CXCR2^+^ neutrophils from entering the tumor, which led to an improved anti-tumor immunity and response to chemotherapy [113]. CAFs, and iCAFs in particular, likely play a role in the recruitment of monocytes into peritumoral areas since they express high levels of CCL2 [33]. CCL2 has also been implicated in attracting myeloid cells to the central nervous system to mediate cachexia [127].

### 5.3. Dual Role of CCR5 in PDAC

The CCR5/CCL5 axis appear to have dual functions in PDAC. Murine models treated with CD40 agonists showed an increased influx of CD4^+^ T cells into tumors, with a concomitant increased response to immunotherapy which was dependent on CCL5 [128]. However, it has also been shown that Tregs generally express higher levels of CCR5 compared to effector T cells and that tumor-derived CCL5 promoted an influx of Tregs, which resulted in increased tumor growth [129]. Singh et al. demonstrated that CCR5 and CCL5 are highly expressed in metastatic human PDAC and that CCL5 promoted proliferation and invasion of tumor cells, suggesting that the CCR5/CCL5 axis is involved in metastasis [130]. It was subsequently shown that CCR5 inhibition led to remission of liver metastasis in a human xenograft model, which was mediated by the downregulation of cell cycle processes in human PDAC cells [131].

### 5.4. The CXCR3 Axis Can Promote T-Cell Infiltration but also Contribute to Chemotherapy Resistance

The chemokines that signal through CXCR3, including CXCL9 and CXCL10, have been suggested to promote T-cell infiltration and activation in melanoma and other solid tumors [42,132,133]. Expression of CXCR3 was necessary for CD8^+^ T-cell anti-tumor responses after treatment with PD-1 inhibitors in mouse models of melanoma [134]. Levels of CXCL9 and CXCL10 were also correlated with the presence of tumor-infiltrating T cells in melanoma patients and migration assays confirmed that these chemokines were critical for T-cell influx [42]. In advanced PDAC, high levels of CXCL9 and CXCL10 in plasma were associated with better survival and response to chemotherapy [135]. A stimulator of interferon genes (STING) agonist promoted expression of CXCR3 ligands in a murine model of PDAC, which led to increased effector T-cell infiltration and a decrease in suppressive immune cells [136]. Conversely, high expression of CXCL10 and CXCR3 in the tumor microenvironment has been shown to be associated with a poor prognosis in human PDAC in several studies [137,138,139,140]. It has been suggested that CXCR3^+^ regulatory T cells are attracted to the tumor microenvironment as a result of intratumoral CXCL10 secretion [139]. Likewise, CXCL9 has been suggested to promote tumor progression by inducing STAT3-dependent suppression of cytotoxic T cells [141]. The disparity between the studies both within pancreatic cancer and between other types of solid tumors such as melanoma is not known, but it is possible that CXCR3 and its ligands affect malignant cells differently in various types of cancer. Indeed, the majority of human PDAC tumors contain a subset of tumor cells expressing CXCR3 and exposure to CXCL10 induced resistance to gemcitabine [138]. Furthermore, the role of CAFs in CXCR3-mediated modulation of tumor immune cells is as yet not known.

## 6. Therapeutic Treatments to Target Chemokines

T-cell infiltration into the tumor nest is crucial for a good prognosis in pancreatic cancer patients. Targeting chemokines may putatively have an impact on the immune profile and enhance the impact of both standard therapies and immunotherapies. As described above, many antagonists have been tested in preclinical animal models. However, only a few are currently being evaluated in clinical trials to treat pancreatic cancer patients. These include blocking of CCR2, CCR5, CXCR2, and CXCR4. Table 2 includes a summary of the completed and active clinical trials targeting chemokine receptors in pancreatic cancer.

### 6.1. Targeting CCL2/CCR2 Chemokine Axis

The safety and the efficacy of CCR2 blockade with PF-04136309, in combination with chemotherapy (folfirinox), has been shown in a phase Ib clinical trial in pancreatic cancer patients with advanced or borderline resectable tumors [114]. The mechanism of action of this molecule is to inhibit the circulation of monocytes from the bone marrow to the tumor. Blockade of the CCL2/CCR2 chemokine axis was well tolerated by the patients, which also showed a partial response. Combination treatment with chemotherapy resulted in a reduction in tumor-associated macrophages and an increased number of CD8^+^ and CD4^+^ T cells in the primary tumor compared to chemotherapy alone [114]. However, another safety and pharmacokinetics/pharmacodynamics phase Ib study which combined PF-04136309 and chemotherapy (gemcitabine/nab-paclitaxel) in patients with metastatic PDAC showed no significant improvement compared to chemotherapy alone but showed possible toxic effects in the lungs [115].

### 6.2. Targeting CCL5/CCR5 Chemokine Axis

Maraviroc is a CCR5 antagonist drug approved by the FDA to treat HIV patients. Preclinical models in pancreatic cancer have shown that inhibition of the CCL5/CCR5 axis with maraviroc leads to tumor cell apoptosis and growth arrest [131]. Clinical trials in colorectal cancer with this drug (NCT01736813, NCT03274804) have shown promising results [142,143], with reduced proliferation of tumor cells and a shift towards M1 macrophages in one of the trials [142]. After these encouraging results, clinical trials with maraviroc combined with immune checkpoint inhibitors are currently ongoing for metastatic pancreatic cancer (NCT04721301). To boost the specific and encouraging effects of CCR2 and CCR5 antagonists, a phase Ib/II clinical trial with dual blockade of CCR2 and CCR5 with BMS 813160 as a monotherapy or in combination with chemotherapy or immunotherapy is currently ongoing for advanced pancreatic cancer patients (NCT03184870) [144].

### 6.3. Targeting CXCR1/2 and Their Ligands Chemokine Axis

Another chemokine antagonist that has been shown to alter the tumor immune environment is the CXCR1/2 antagonist SX-682. The main function of CXCR2 is to regulate the recruitment and migration of neutrophils and MDSCs. SX-682 has been shown to enhance Th1 immune response in several animal models including melanoma, breast, lung, and prostate cancer [145,146,147]. This inhibitor is currently undergoing a safety evaluation in a phase I clinical trial for pancreatic cancer patients in combination with anti-PD-1 treatment (NCT04477343).

### 6.4. Targeting CXCL12/CXCR4 Chemokine Axis

The CXCL12/CXCR4 axis excludes effector T cells from the tumor nests, impacting the efficacy of immune checkpoint inhibitors. The administration of the CXCR4 antagonist AMD3100 induced CD8^+^ T cells infiltration and promoted a rapid activation and response of intratumoral T cells, natural killer cells, and B cells in a phase I clinical trial for metastatic PDAC [118]. The safety and clinical benefit of AMD3100 combined with anti-PD-1 treatment is being assessed in a phase II clinical trial (NCT04177810). A phase I/II clinical trial with the CXCL12 inhibitor NOX-A12 in combination with PD-1 inhibition showed an increased immune response in approximately half of the patients [119]. Similarly, another phase II clinical trial targeting CXCR4 with BL-804 together with PD-1 inhibitors showed an increased T-cell infiltration and enhanced CD8^+^ T-cell cytotoxicity [120]. The study also showed a decrease in intratumoral MDSCs and circulating Tregs with a modest increase in overall survival [120]. Other ongoing phase II clinical trials targeting both CXCR4 with BL-804 and PD-1 will determine whether immunotherapy combined with chemokine blockade can rescue the patients’ anti-tumor immunity (NCT02907099, NCT04543071).

## 7. Conclusions

Pancreatic CAFs have emerged as important regulators of the tumor microenvironment, both as restrainers of tumor growth but also as suppressors of tumor-reactive immunity. The recent discoveries about the diverse functions of different CAF subpopulations have significantly increased our understanding of the complex pancreatic stroma, but many questions still remain. The low mutational burden and the suppressive milieu in pancreatic cancer have been suggested to contribute to the lack of response to immune checkpoint inhibitors, but a key issue may be to assist T cells to efficiently come within close proximity of the malignant cells. Several lines of evidence suggest that chemokines and their cognate ligands play an important role in promoting T-cell exclusion from the tumor and further preclinical and clinical studies evaluating the role of chemokines are necessary to take full advantage of immune checkpoint therapeutics.

## Data Availability

Not applicable.
